# Supplementary figures and images for: A DCL3 dicing code within Pol IV-RDR2 transcripts diversifies the siRNA pool guiding RNA-directed DNA methylation
Source: eLife. 2022 Jan 31;11:e73260. doi: 10.7554/eLife.73260 (PMC8846587; doi:10.7554/eLife.73260)

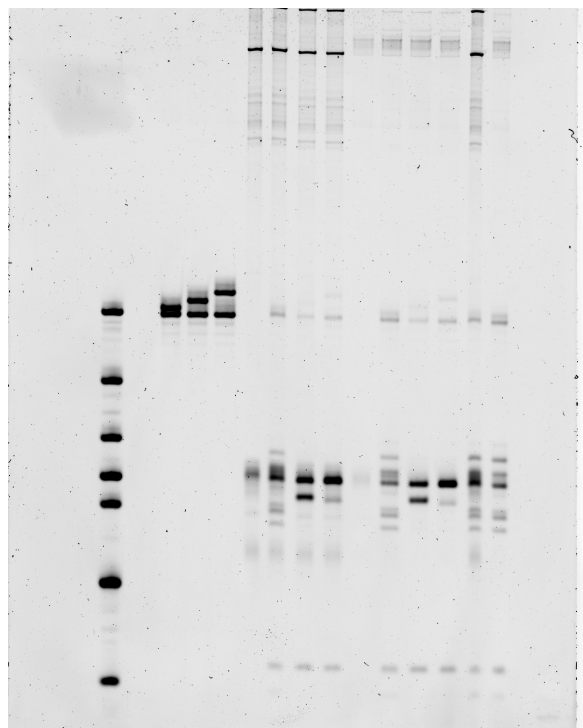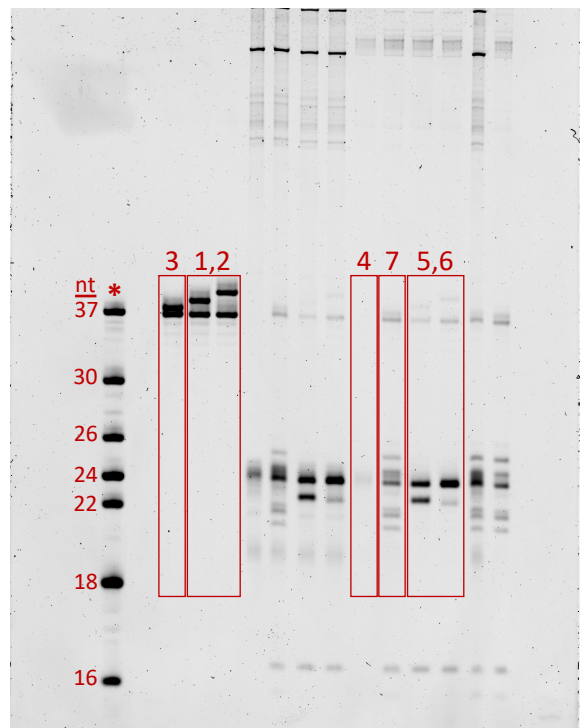

Supplement: Figure 1—source data 1. — Raw gel images are provided next to annotated gel images showing the portions of the images used in the various panels of Figure 1C. The asterisk denotes a lanes containing size markers. Numbers indicate the lanes of Figure 1C. The images show SYBR Gold-stained RNAs resolved by denaturing polyacrylamide gel electrophoresis (PAGE) and imaged using a Bio-Rad Laboratories ChemiDoc MP Imaging System. [file elife-73260-fig1-data1.pdf]

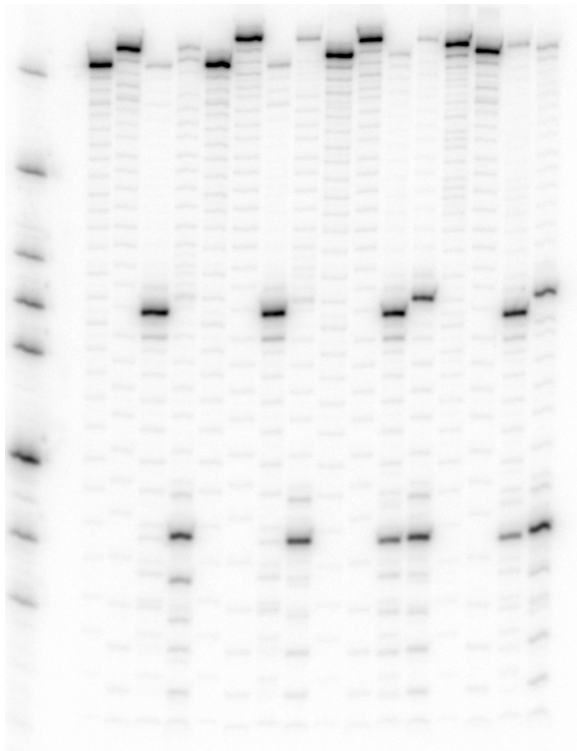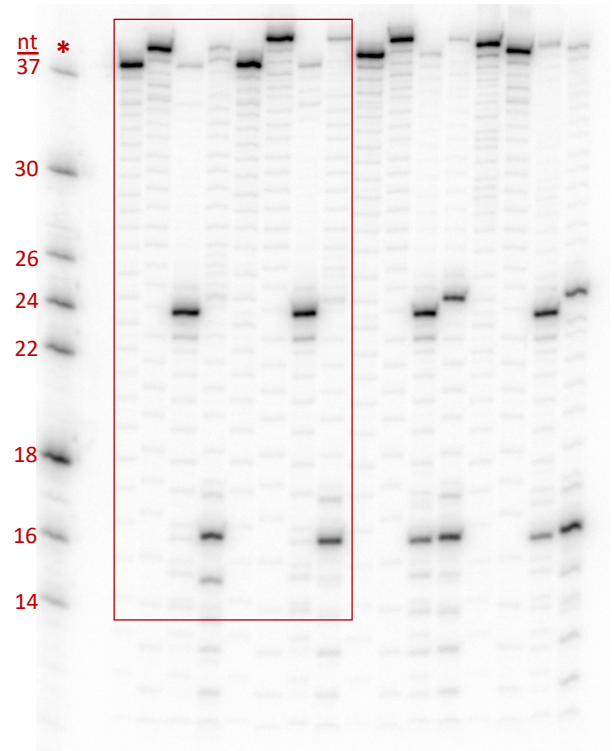

Supplement: Figure 1—source data 2. — Raw gel images are provided next to annotated gel images showing the portions of the images used in the various panels of Figure 1D. The asterisk denotes a lane containing size markers. The images were obtained by phosphorimaging of dried polyacrylamide gels on which 32P-labeled RNA species were resolved by denaturing PAGE. [file elife-73260-fig1-data2.pdf]

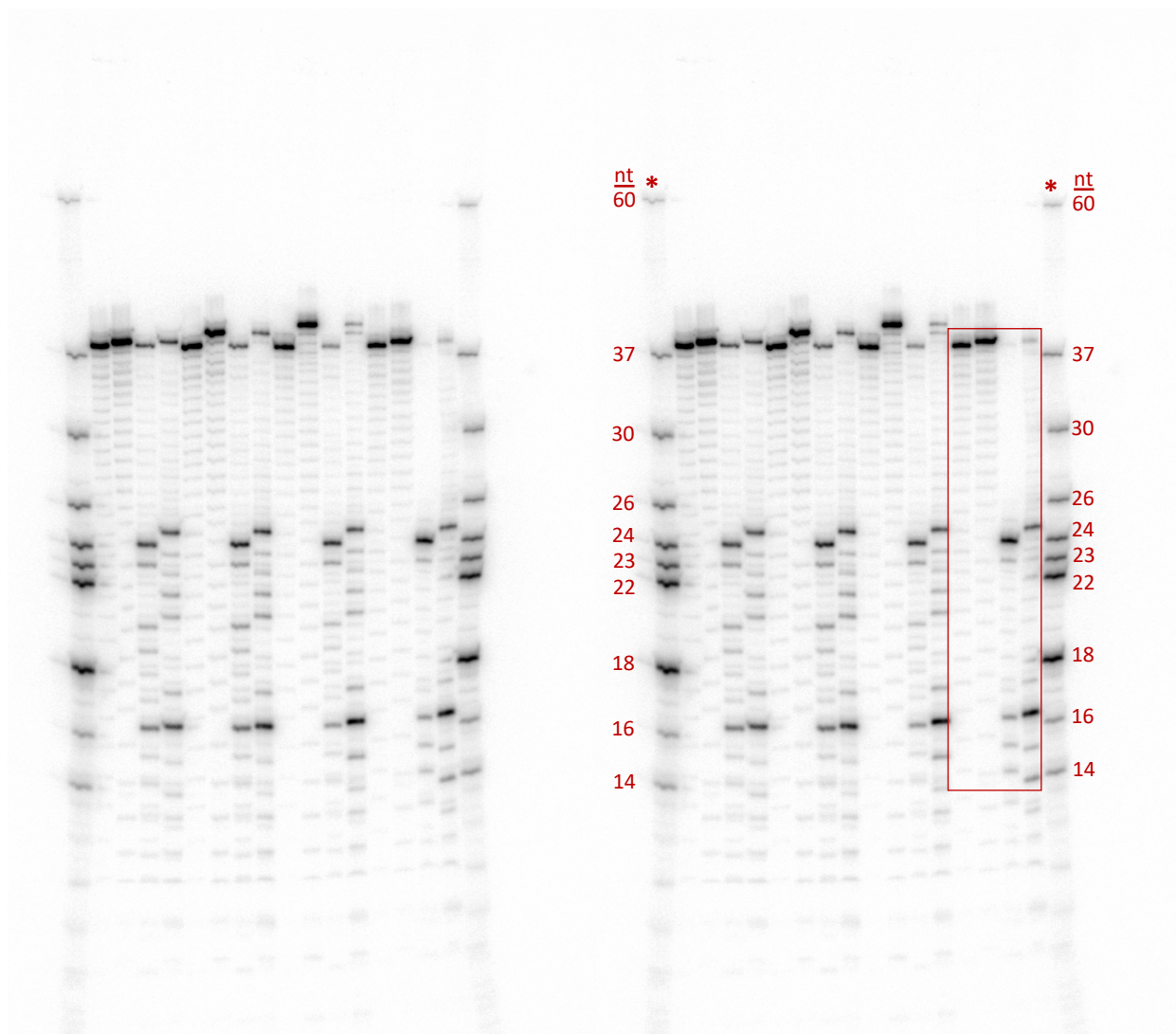

Supplement: Figure 1—source data 3. — Raw gel images are provided next to annotated gel images showing the portions of the images used in the various panels of Figure 1E. Asterisks denote lanes containing size markers. The images were obtained by phosphorimaging of dried polyacrylamide gels on which 32P-labeled RNA species were resolved by denaturing PAGE. [file elife-73260-fig1-data3.pdf]

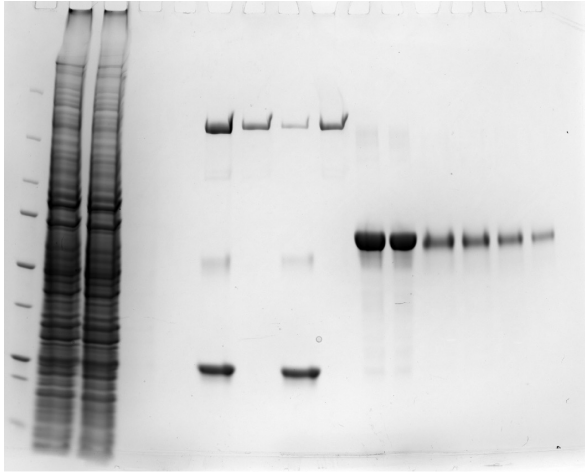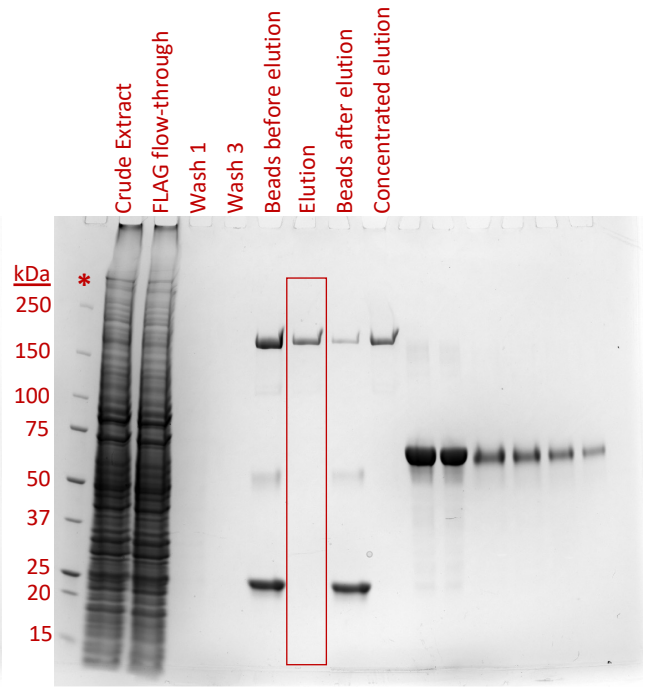

Supplement: Figure 1—figure supplement 1—source data 1. — Duplicate digital images of a 4%–15% gradient SDS-PAGE gel stained with Coomassie Brilliant Blue. The red rectangle in the image on the right shows the portion of the raw image used in Figure 1—figure supplement 1A. The text above the right image indicates the fractions obtained during affinity purification of DCL3. The lane marked by an asterisk includes protein standards, with masses indicated in kilodaltons (kDa). [file elife-73260-fig1-figsupp1-data1.pdf]

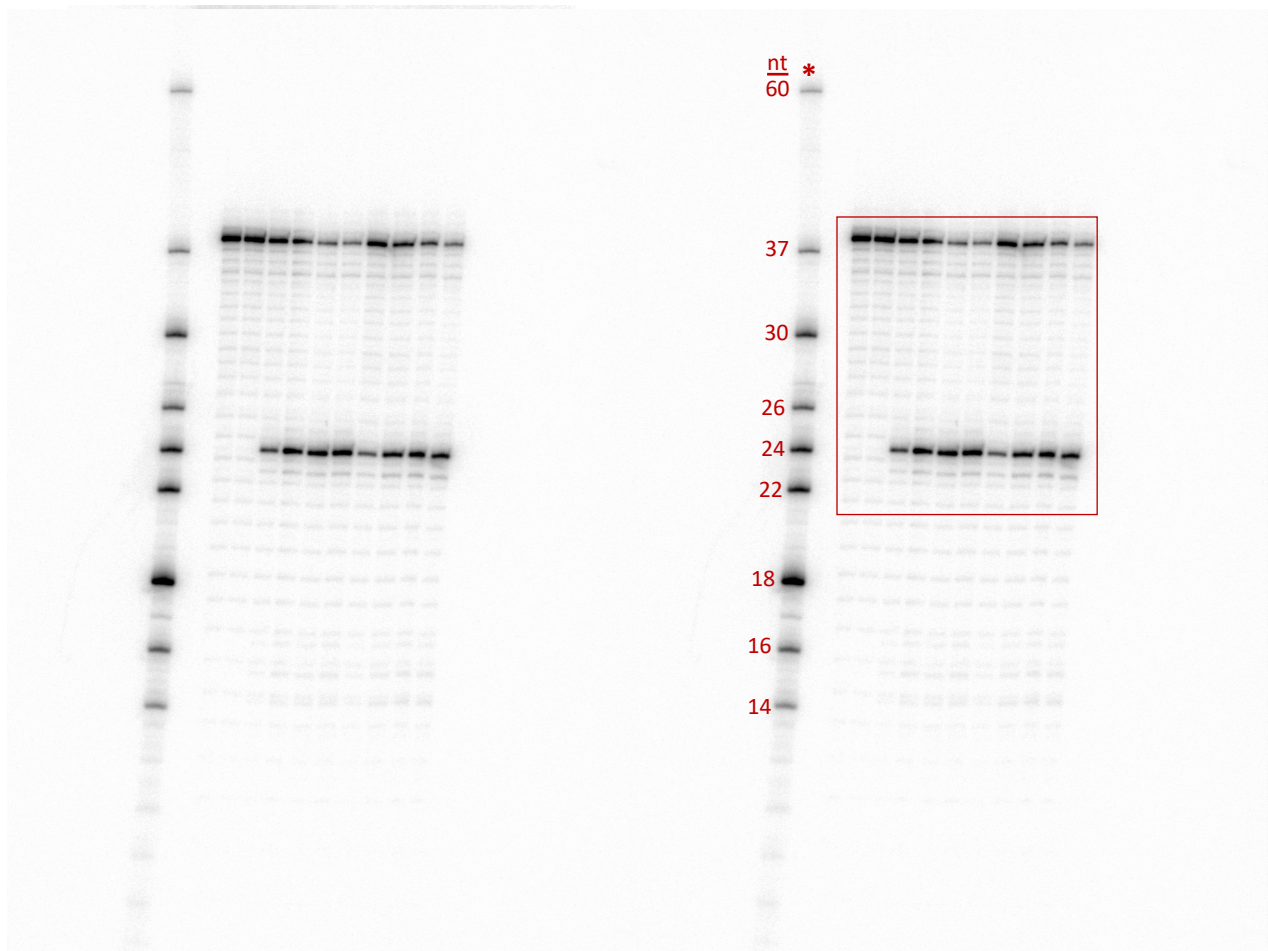

Supplement: Figure 1—figure supplement 1—source data 2. — Duplicate images obtained by phosphorimaging of a dried denaturing PAGE gel on which 32P-labeled RNA species were resolved. The red rectangle in the image on the right shows the portion of the raw image used in Figure 1—figure supplement 1B. The lane marked by an asterisk contains RNA size standards whose lengths are shown in nucleotides (nt). [file elife-73260-fig1-figsupp1-data2.pdf]

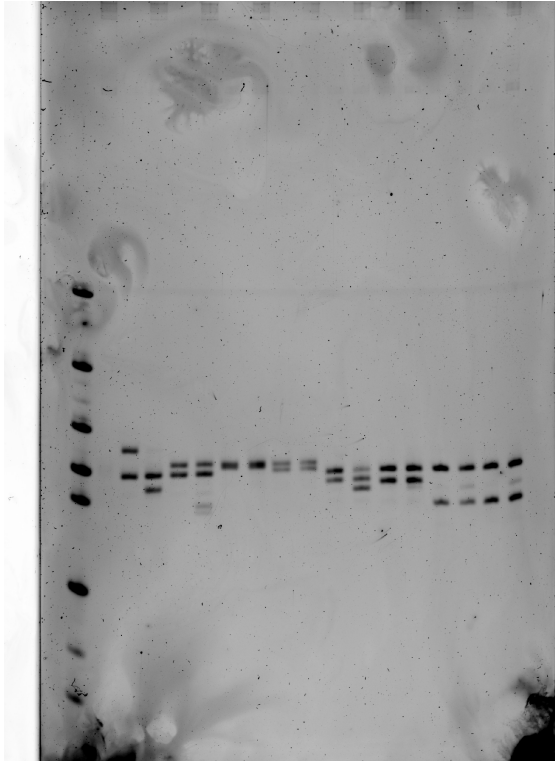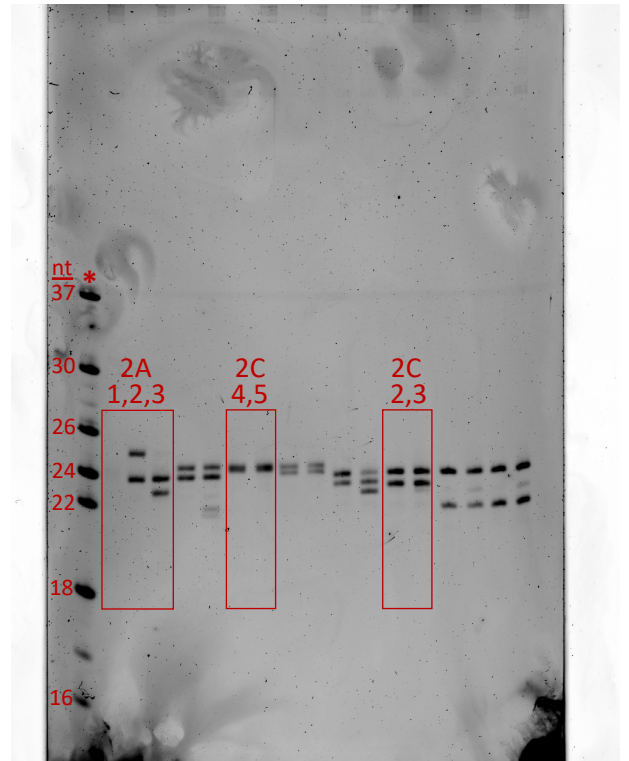

Supplement: Figure 2—source data 1. — Duplicate images of a denaturing PAGE gel with RNA species stained with SYBR Gold. The red rectangles in the image on the right show the portions of the raw image used in different lanes of Figure 2 panels A or C, as indicated. Note that lane 1 of Figure 2A is the same as lane 1 of Figure 2C. [file elife-73260-fig2-data1.pdf]

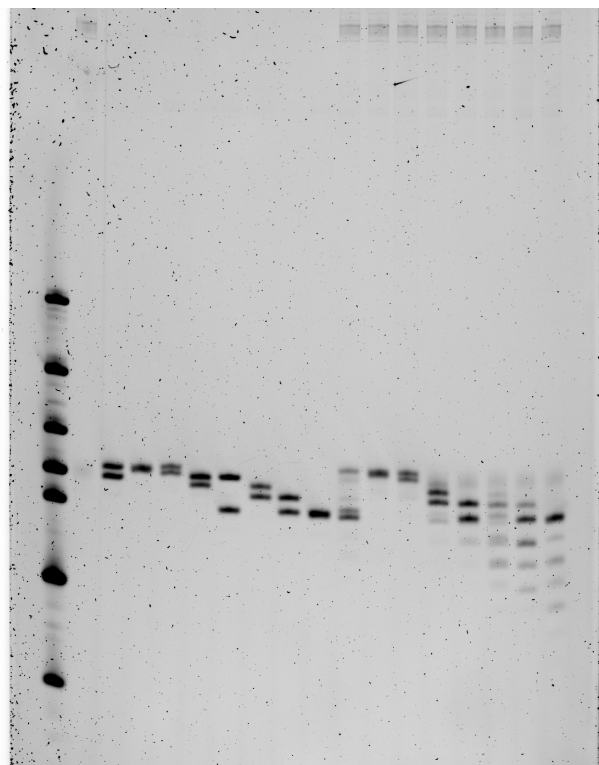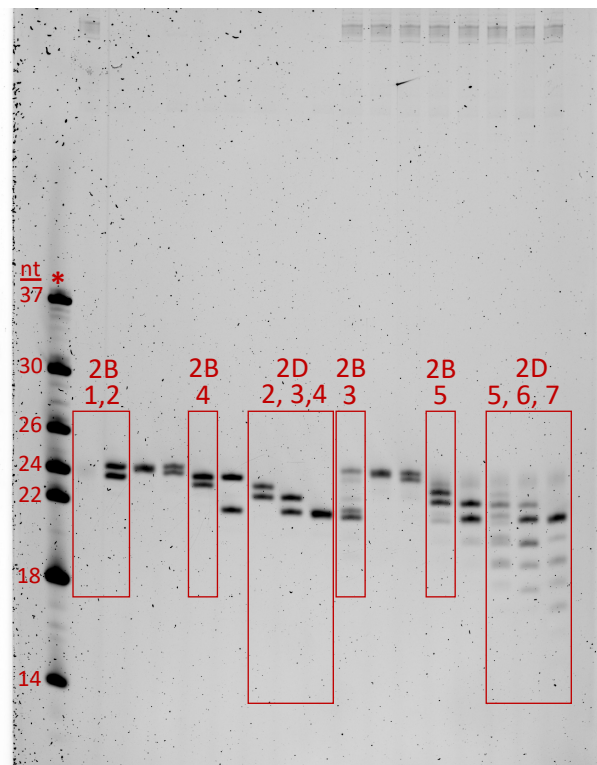

Supplement: Figure 2—source data 2. — Duplicate digital images of a denaturing PAGE gel showing RNA species stained with SYBR Gold. The red rectangles in the image on the right show the portions of the raw image used in lanes of Figure 2 panels B or D, as indicated. Lane 1 of Figure 2B is also lane 1 of Figure 2D. The lane marked by an asterisk contains RNA size standards, with lengths shown in nucleotides (nt). [file elife-73260-fig2-data2.pdf]

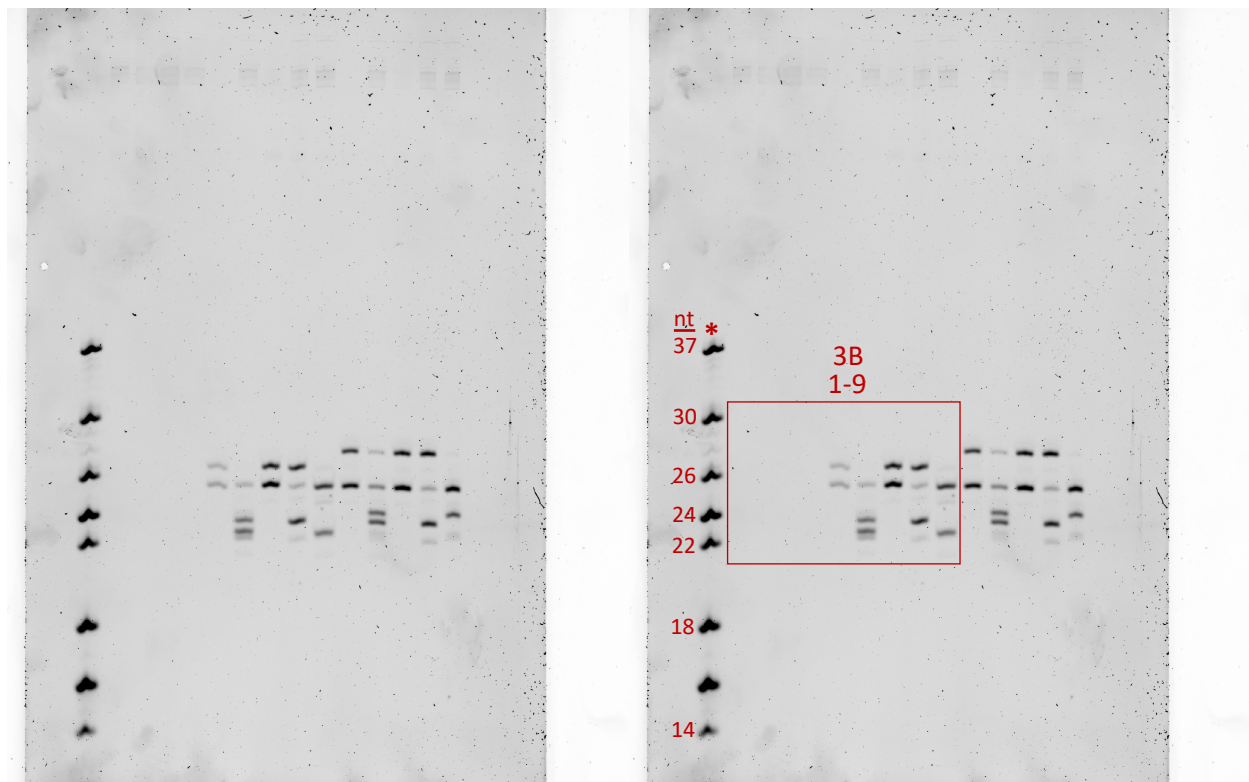

Supplement: Figure 3—source data 1. — Duplicate digital images of a denaturing PAGE gel with RNA species stained with SYBR Gold. The red rectangle in the image on the right shows the portions of the raw image used in Figure 3B, with corresponding lane numbers. The lane marked by an asterisk contains RNA size standards whose length is shown in nucleotides (nt). [file elife-73260-fig3-data1.pdf]

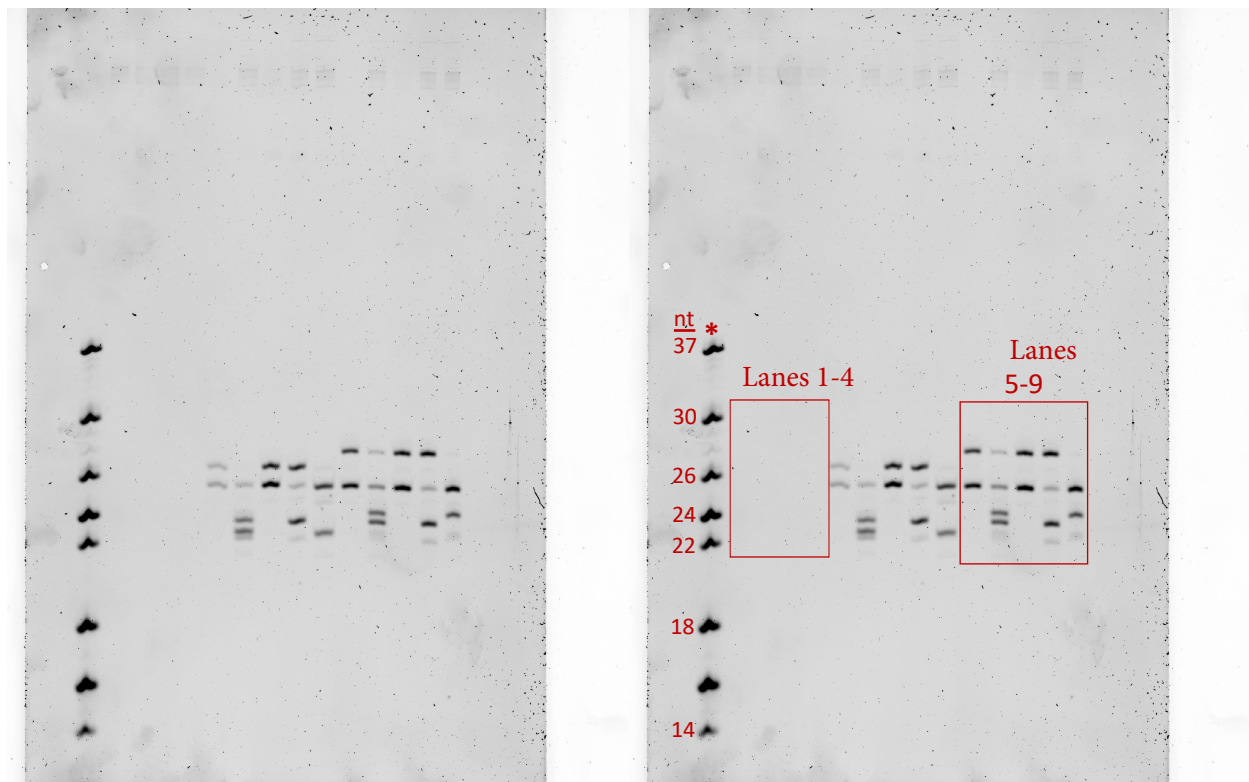

Supplement: Figure 3—figure supplement 1—source data 1. — Duplicate digital images of a denaturing PAGE gel showing RNA species stained with SYBR Gold. The red rectangles in the image on the right show the portions of the raw image used in Figure 3—figure supplement 1, with the corresponding lane numbers. The lane marked by an asterisk contains RNA size standards whose length is shown in nucleotides (nt). [file elife-73260-fig3-figsupp1-data1.pdf]

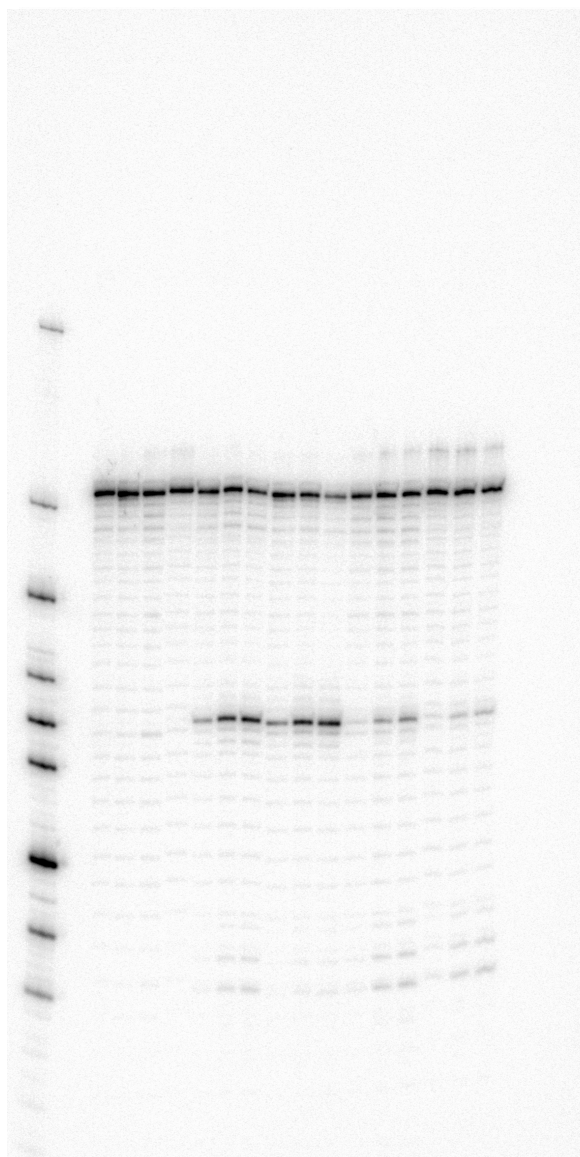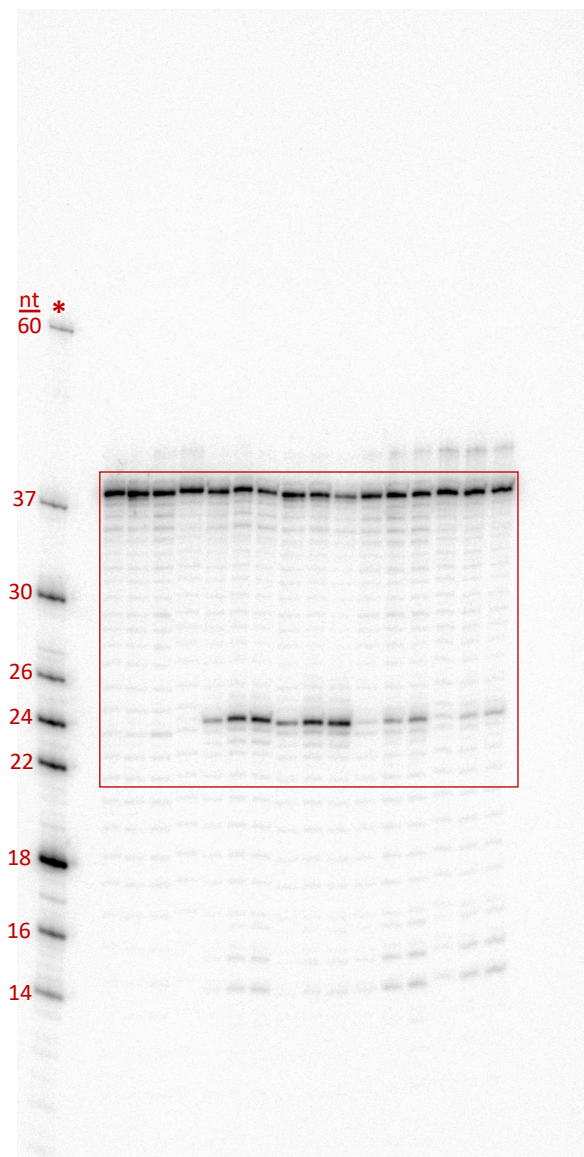

Supplement: Figure 4—source data 1. — Duplicate digital images obtained by phosphorimaging of 32P-labeled RNAs resolved by denaturing PAGE are shown, with red rectangles showing the portion of the raw image used in Figure 4A. [file elife-73260-fig4-data1.pdf]

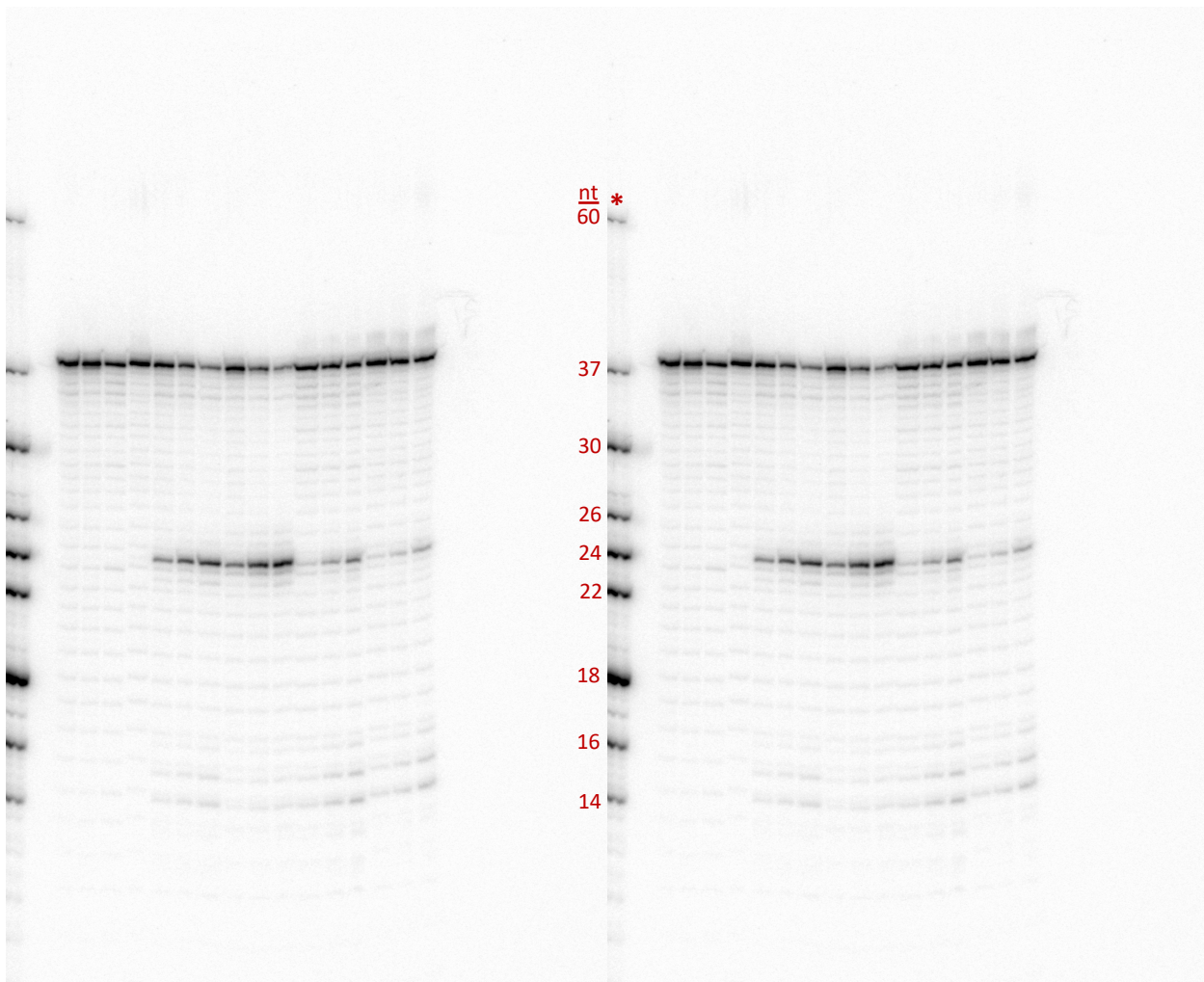

Supplement: Figure 4—source data 2. [file elife-73260-fig4-data2.pdf]

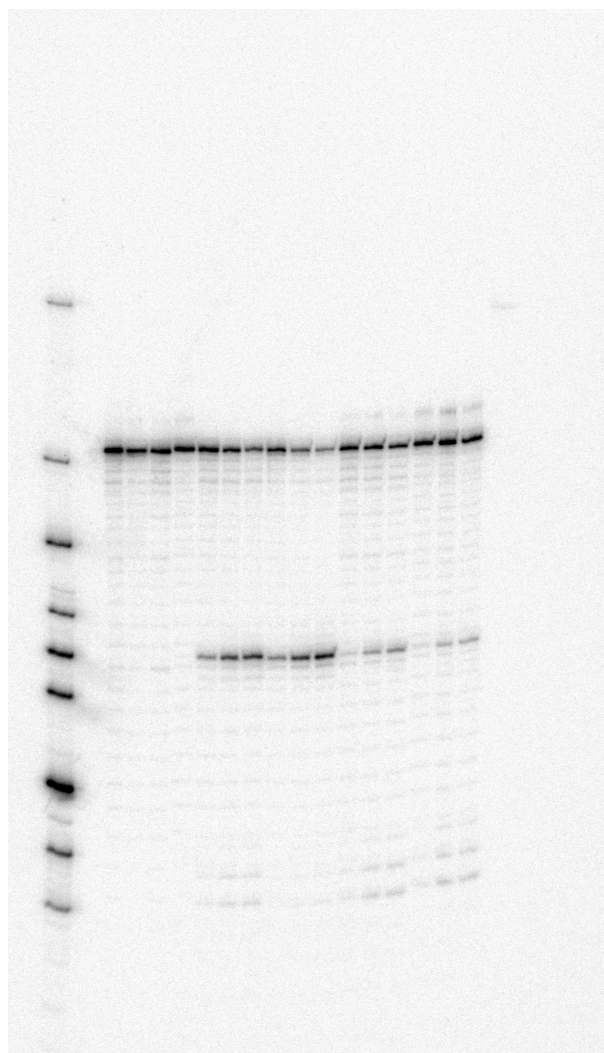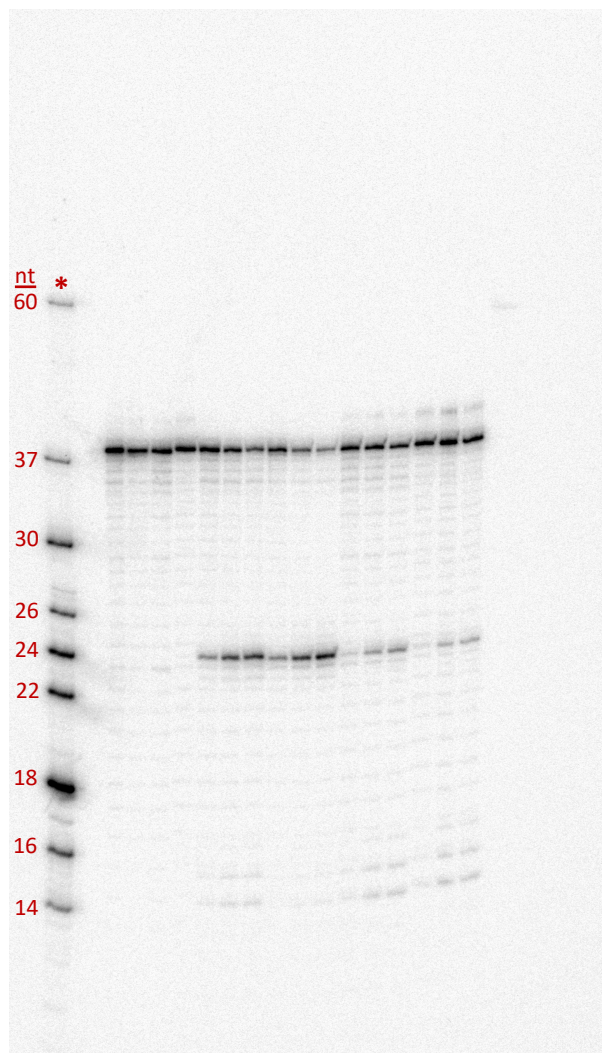

Supplement: Figure 4—source data 3. [file elife-73260-fig4-data3.pdf]

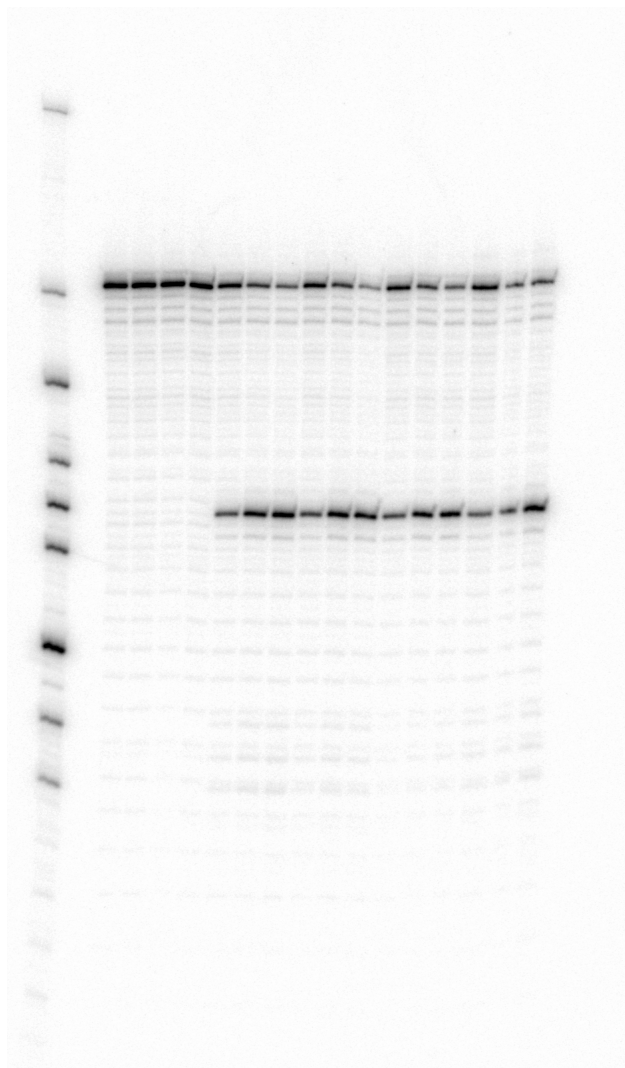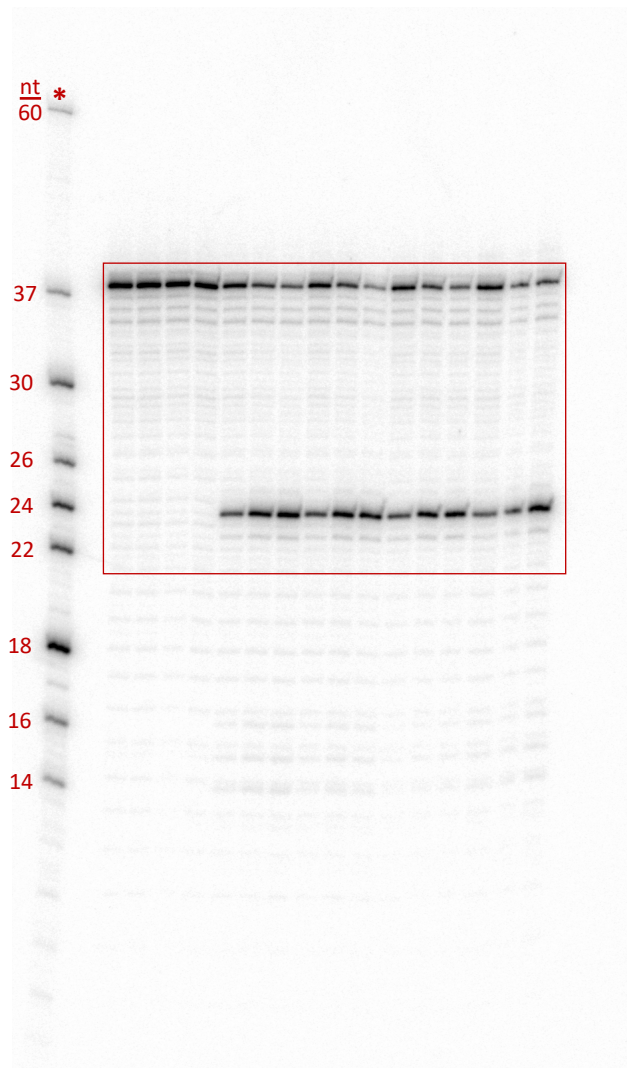

Supplement: Figure 4—source data 5. — Duplicate digital images obtained by phosphorimaging of 32P-labeled RNA resolved by denaturing PAGE are shown, with red rectangles showing the portion of the raw image used in Figure 4B. [file elife-73260-fig4-data5.pdf]

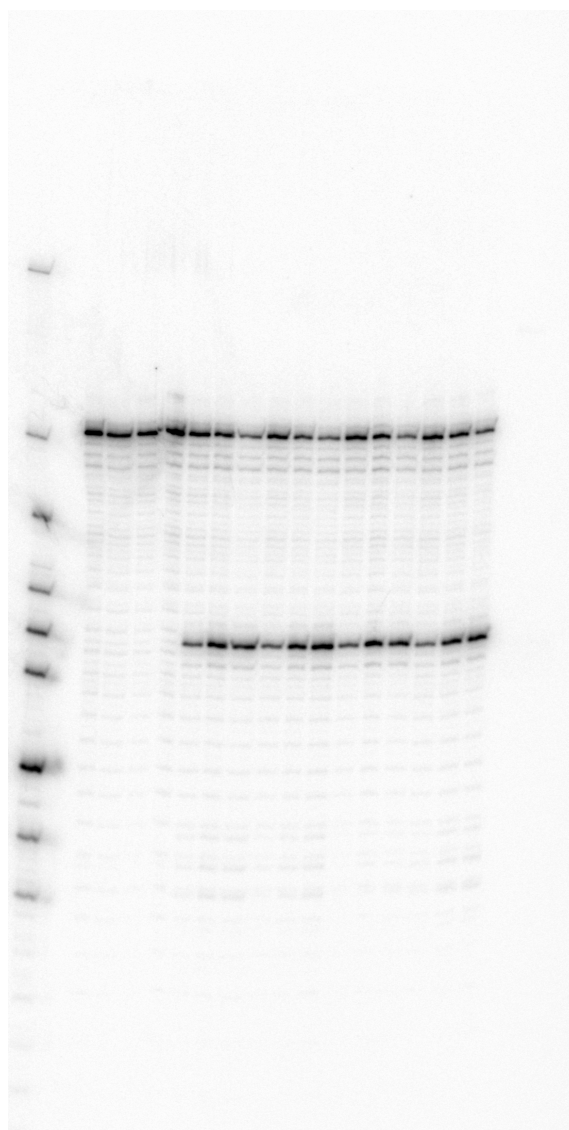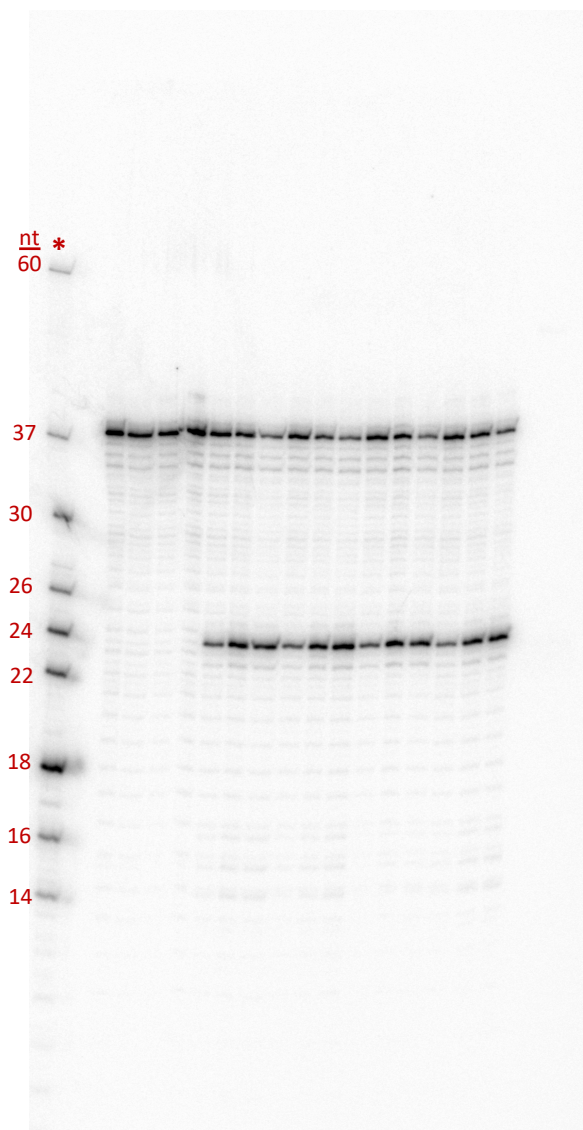

Supplement: Figure 4—source data 6. [file elife-73260-fig4-data6.pdf]

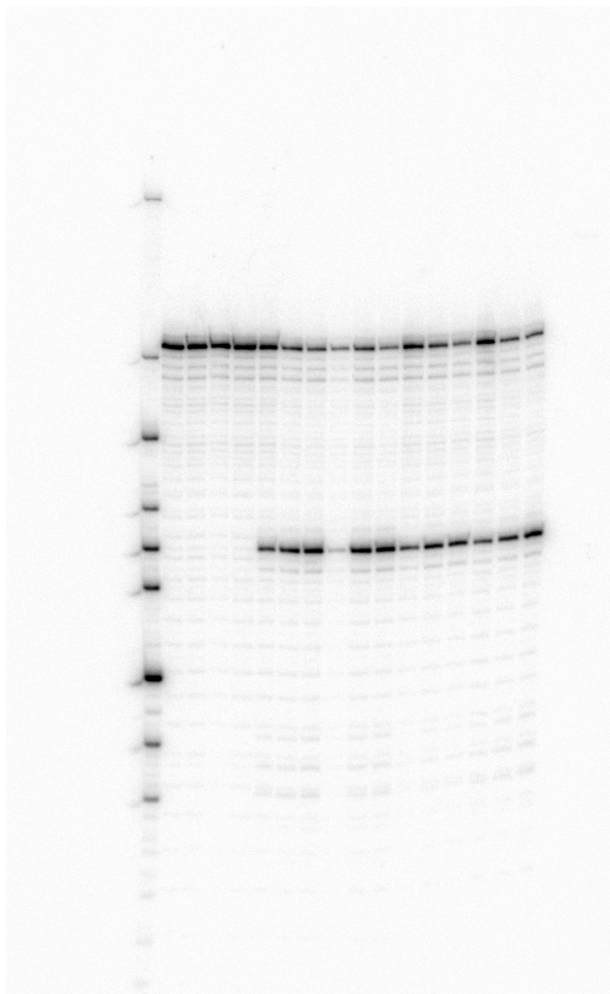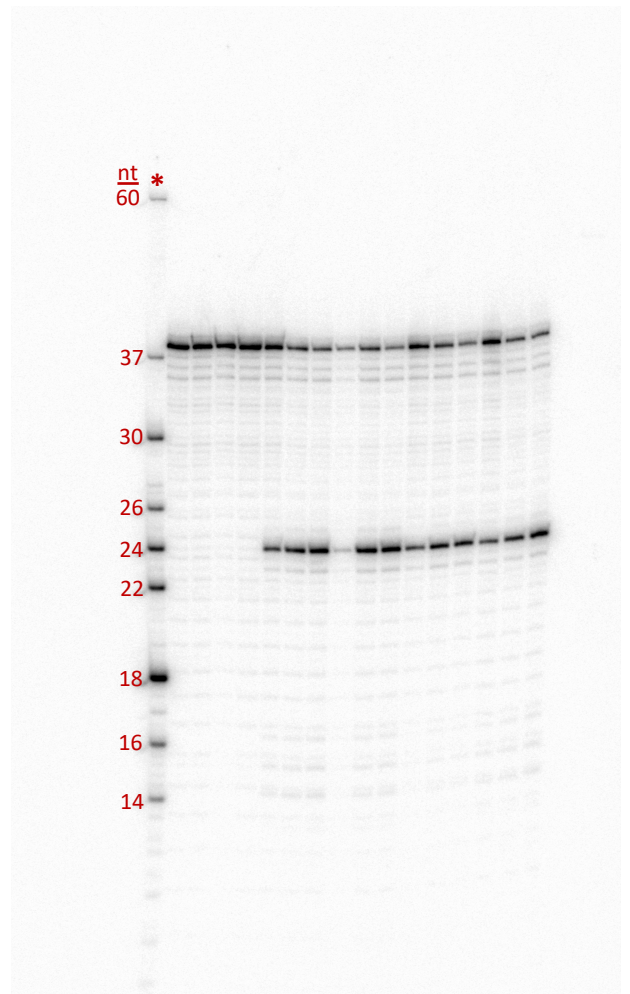

Supplement: Figure 4—source data 7. [file elife-73260-fig4-data7.pdf]

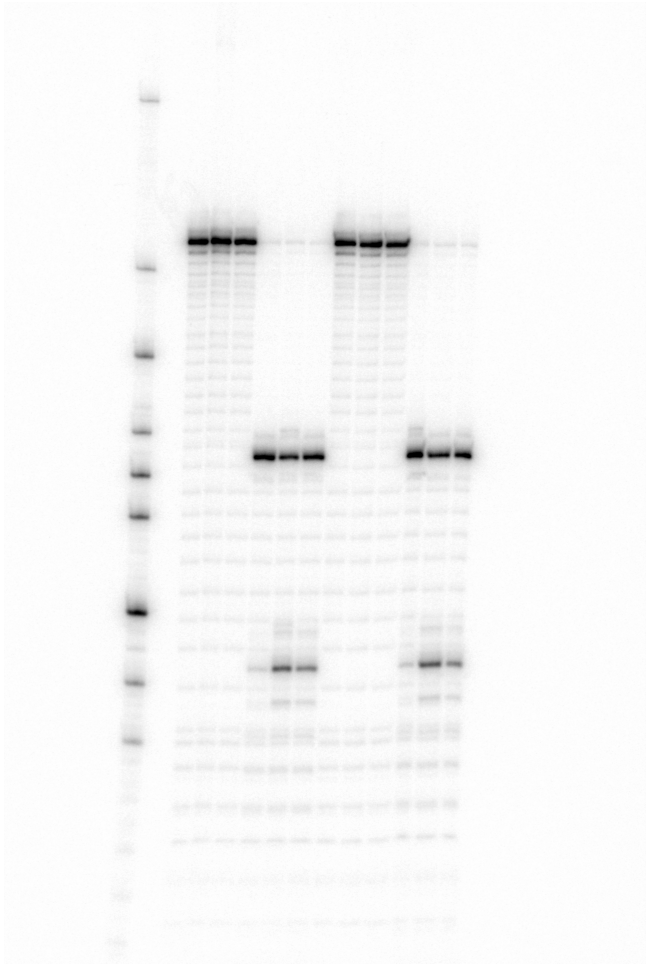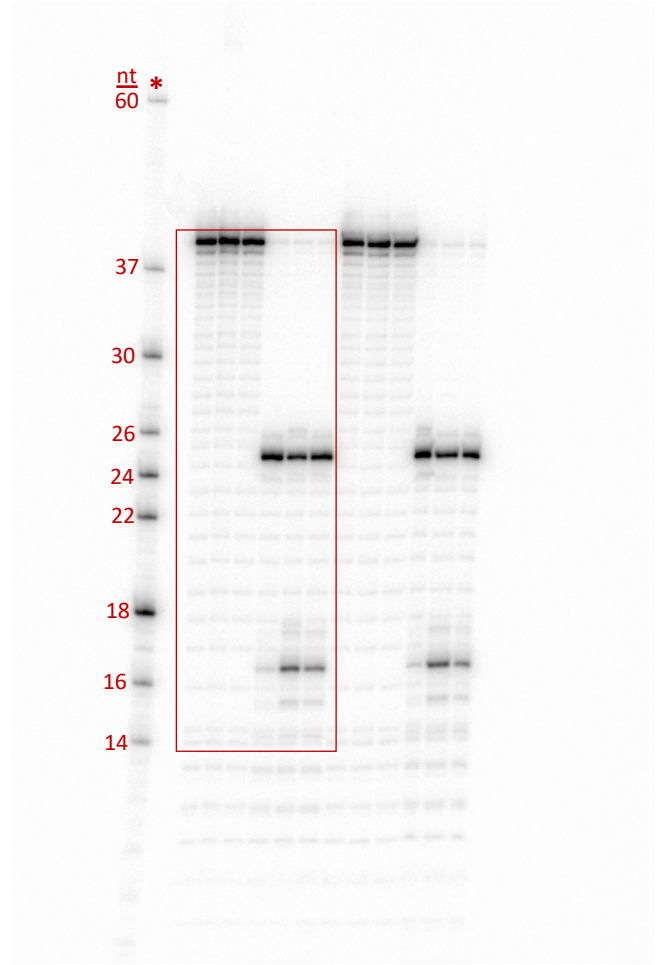

Supplement: Figure 4—source data 9. — Duplicate gel images obtained by phosphorimaging of 32P-labeled RNA resolved by denaturing PAGE. The red rectangle in the image on the right shows the portion of the raw image used in the figure. [file elife-73260-fig4-data9.pdf]

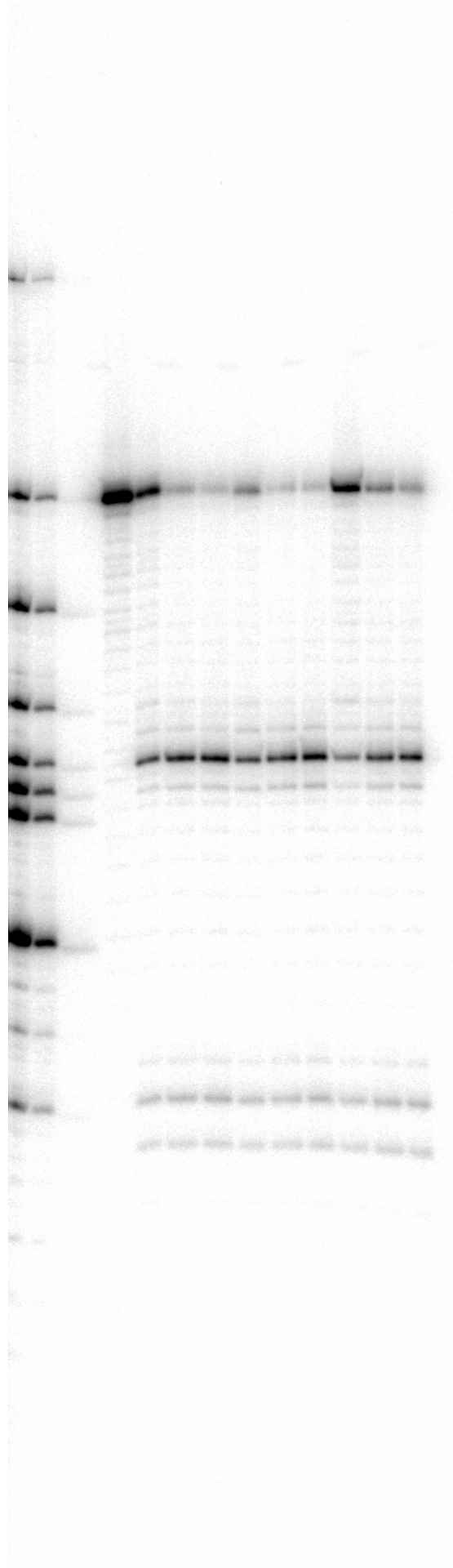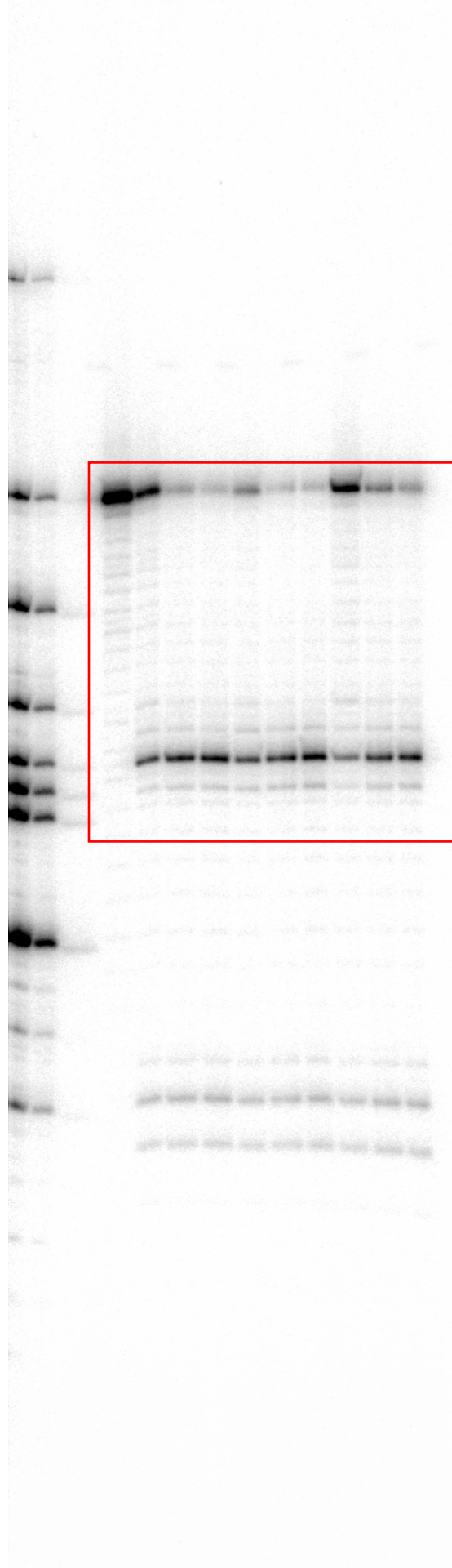

Supplement: Figure 5—source data 1. — Gel images were obtained by phosphorimaging of 32P-labeled RNAs resolved by denaturing PAGE. [file elife-73260-fig5-data1.pdf]

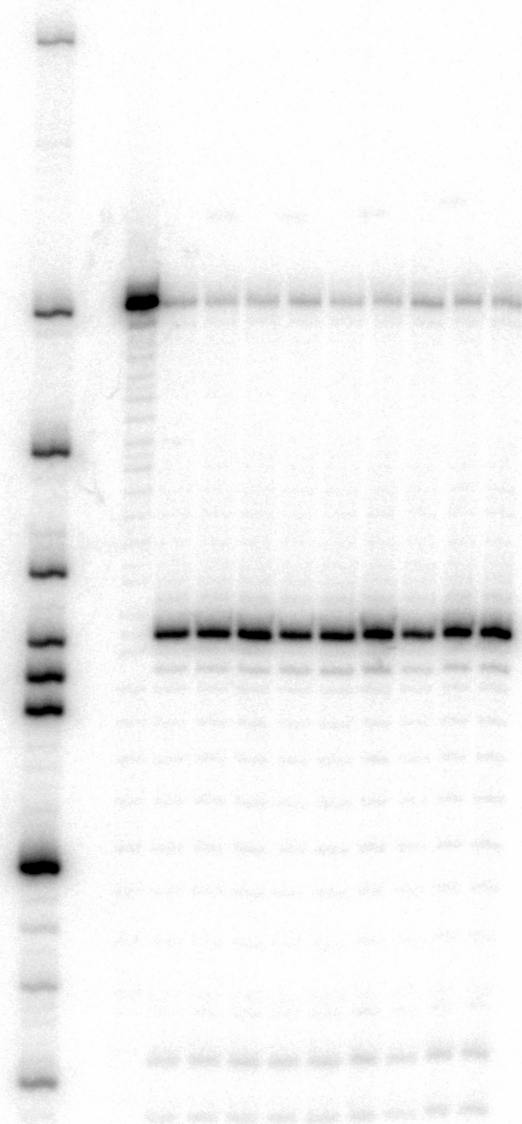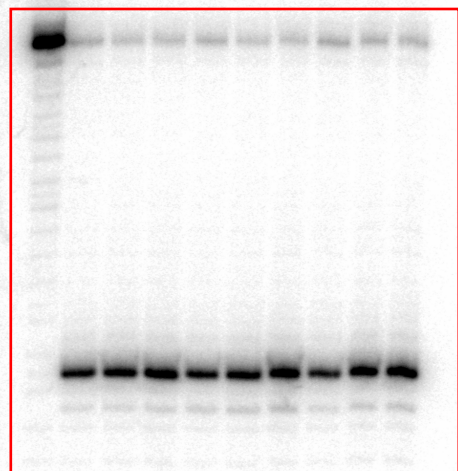

Supplement: Figure 5—source data 2. — Gel images were obtained by phosphorimaging of 32P-labeled RNAs resolved by denaturing PAGE. [file elife-73260-fig5-data2.pdf]

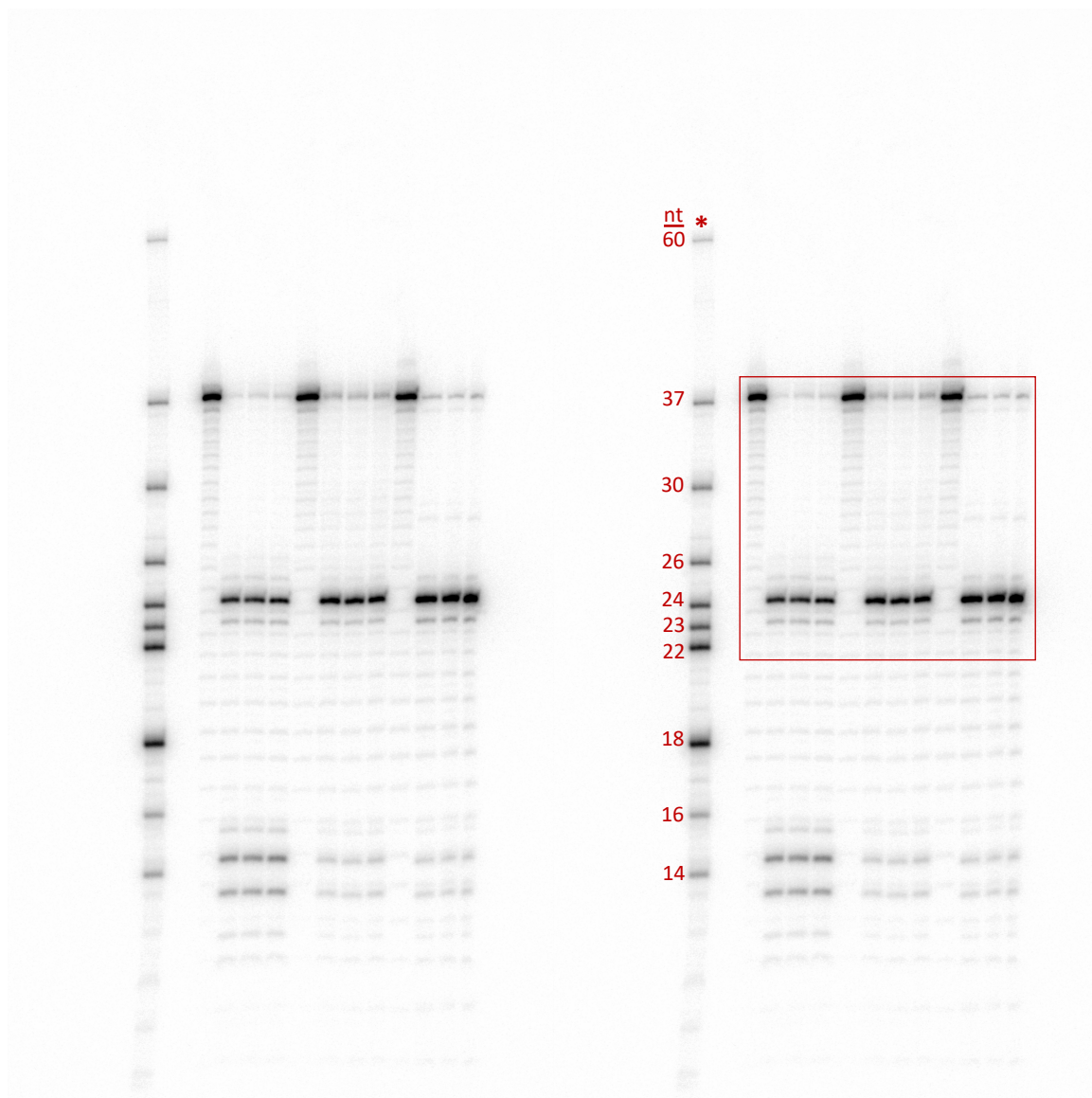

Supplement: Figure 5—figure supplement 1—source data 1. — The gel image was obtained by phosphorimaging of 32P-labeled RNAs resolved by denaturing PAGE. [file elife-73260-fig5-figsupp1-data1.pdf]

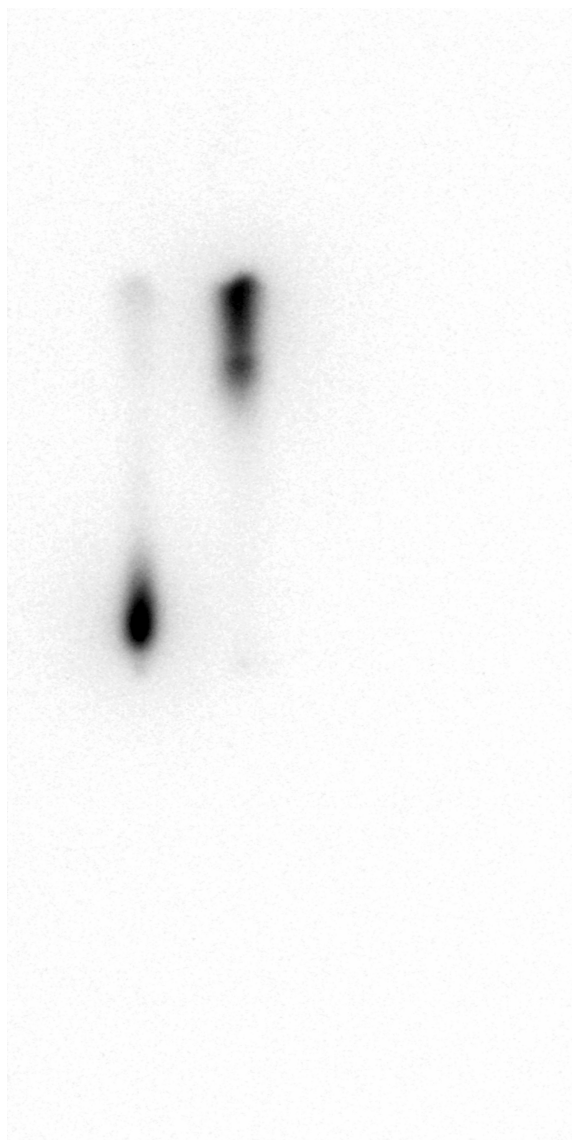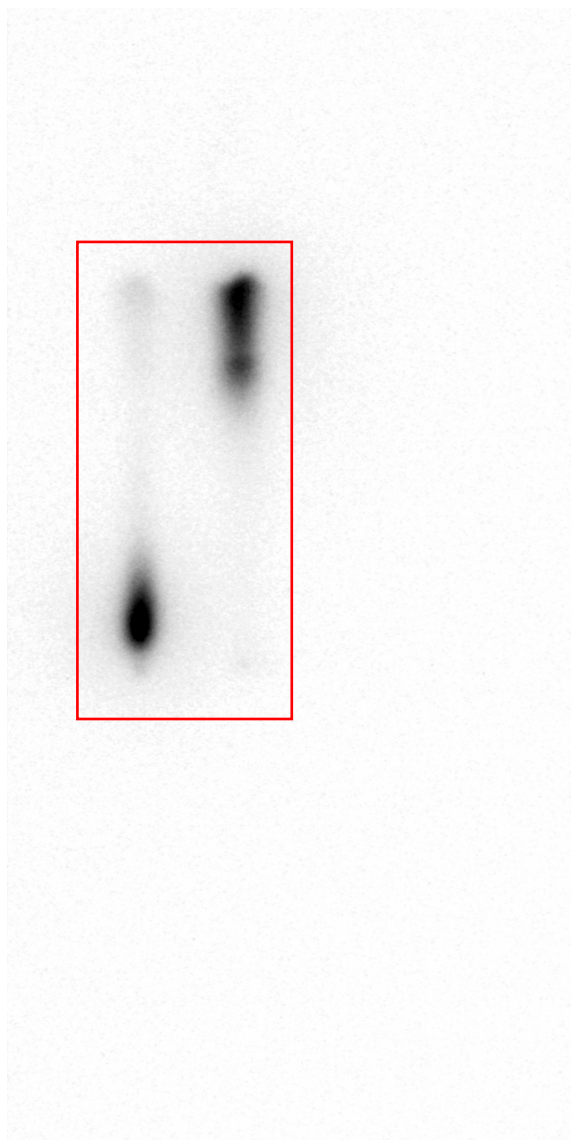

Supplement: Figure 5—figure supplement 2—source data 1. [file elife-73260-fig5-figsupp2-data1.pdf]

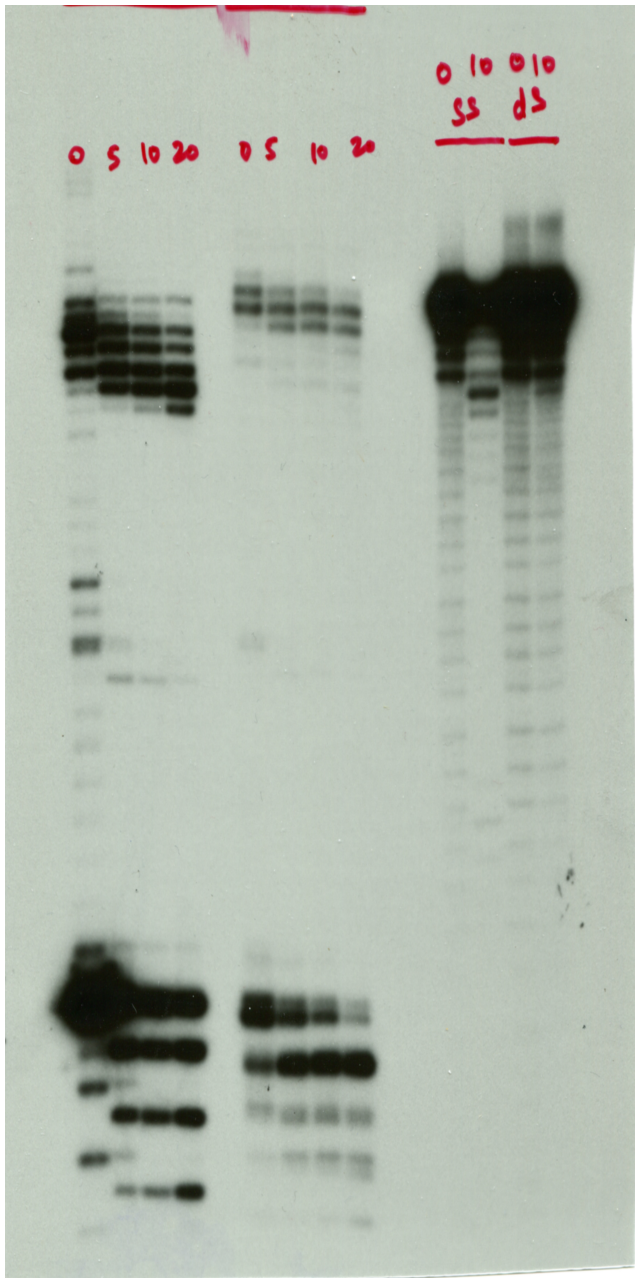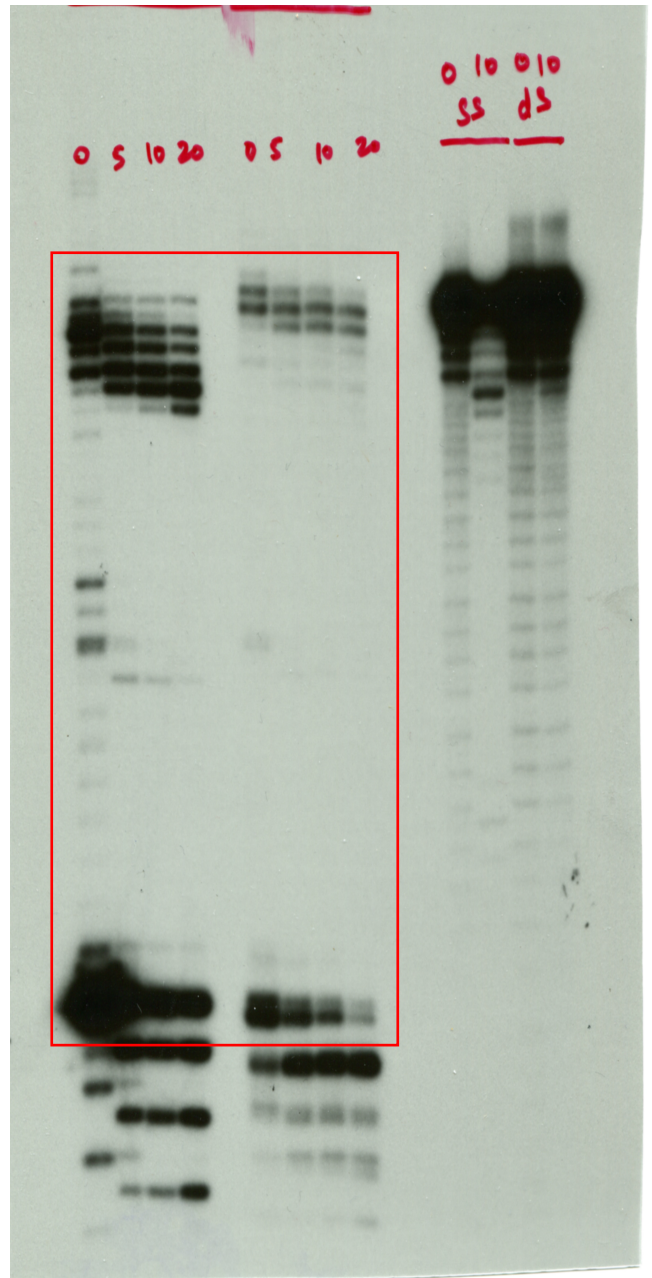

Supplement: Figure 6—source data 1. — The image is that of a sheet of X-ray filmdeveloped after exposure to a dried denaturing PAGE gel on which 32P- labelledRNA species were resolved. [file elife-73260-fig6-data1.pdf]

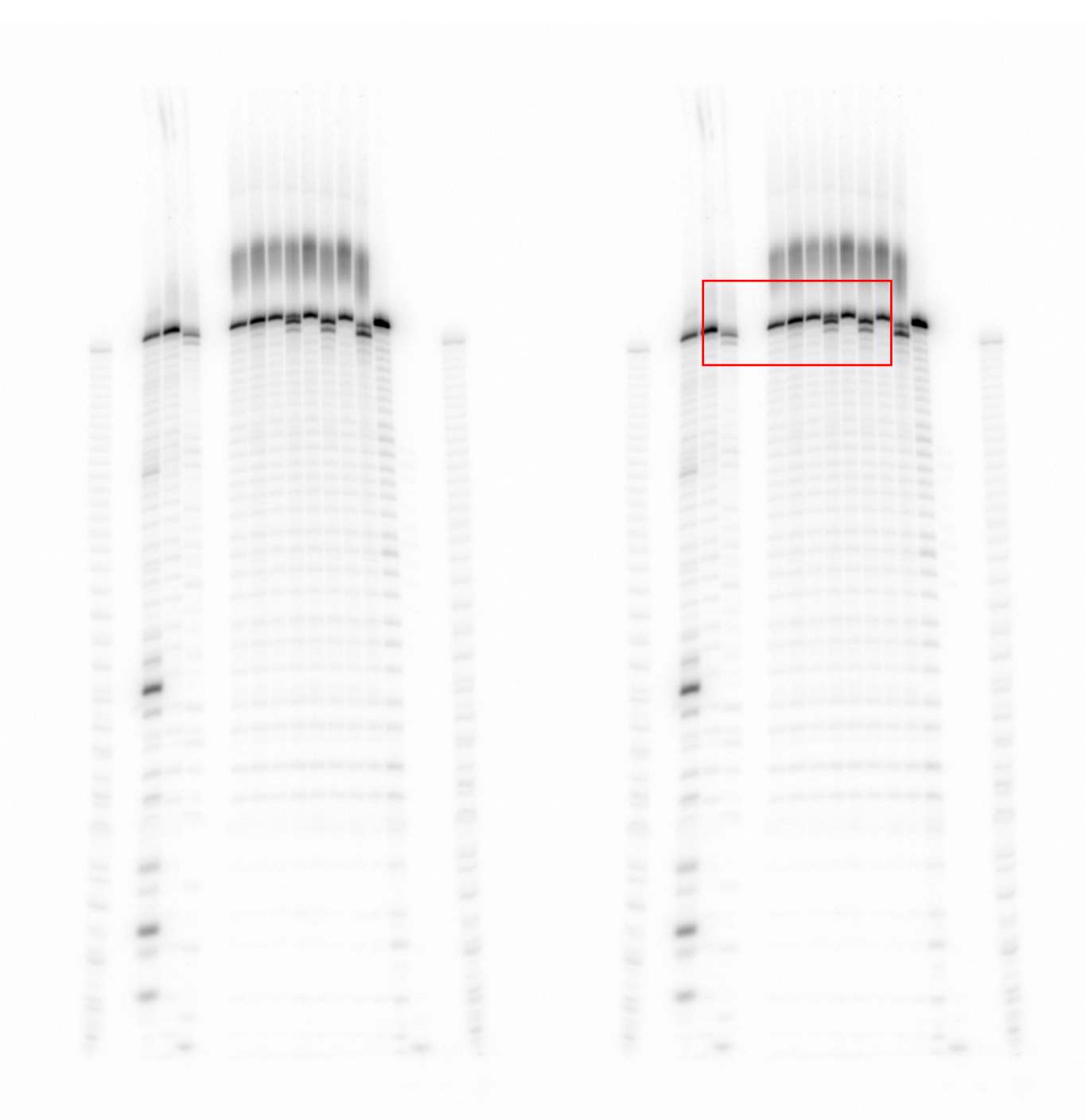

Supplement: Figure 6—source data 2. — Duplicate images were obtained by phosphorimaging of a dried polyacrylamide gel on which 32P- labelled RNA species were resolved by denaturing gel electrophoresis. The red rectangle in the image on the right shows the portion of the raw image used in the figure. [file elife-73260-fig6-data2.pdf]

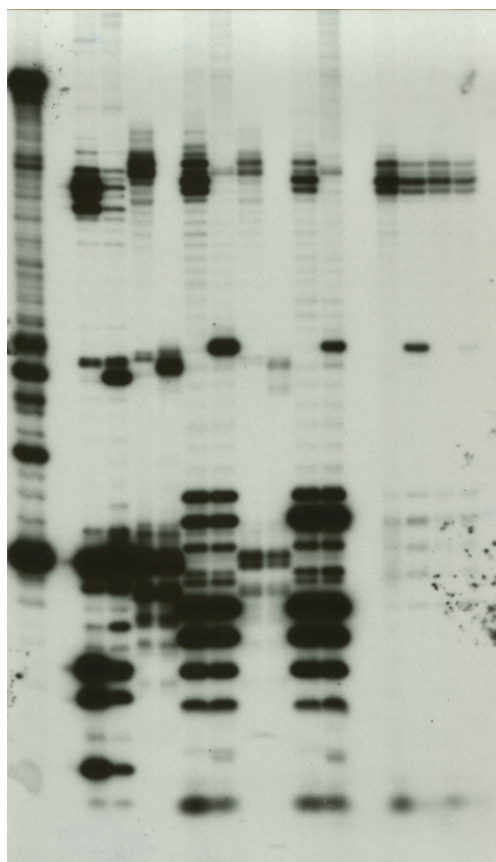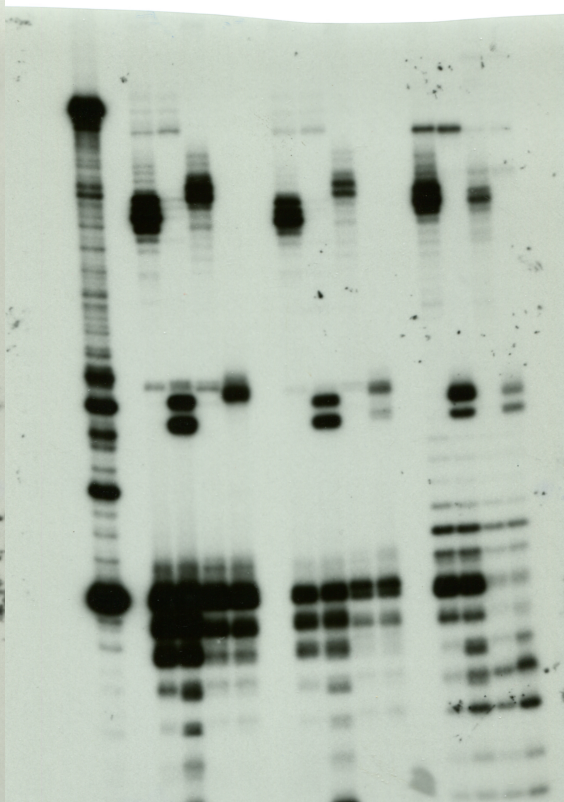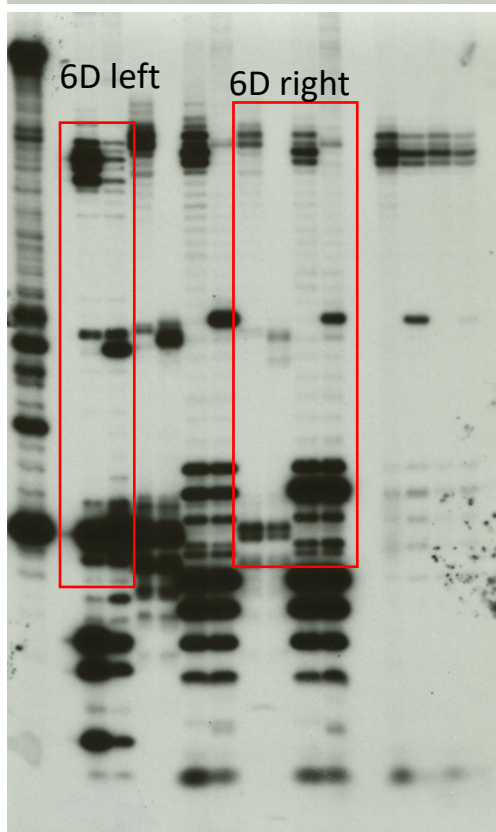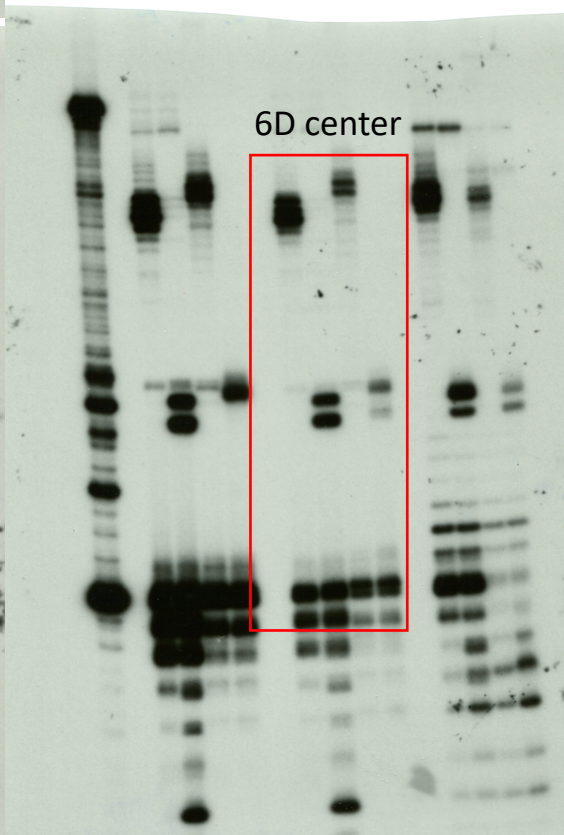

Supplement: Figure 6—source data 3. — In the top and bottom rows are duplicated images of two X-ray films developed after exposure to dried polyacrylamide gels on which 32P- labelled RNA species were resolved by denaturing gel electrophoresis. In the top and bottom rows are duplicated images of two X-ray films developed after exposure to dried polyacrylamide gels on which 32P- labelled RNA species were resolved by denaturing gel electrophoresis. The red rectangles in the bottom row show the groups of lanes used in the left, central and right portions of Figure 6D. [file elife-73260-fig6-data3.pdf]
