# Supplementary material for: A DCL3 dicing code within Pol IV-RDR2 transcripts diversifies the siRNA pool guiding RNA-directed DNA methylation
Source: eLife. 2022 Jan 31;11:e73260. doi: 10.7554/eLife.73260 (PMC8846587; doi:10.7554/eLife.73260)
Supplement: Supplementary file 1. — (a) RNA oligonucleotides used for DCL3 dicing assays. (b) Oligonucleotides used for Pol IV and RDR2 transcription assays. [file elife-73260-supp1.docx]

**Supplementary File 1. Oligonucleotides used in the study**

**Supplementary File 1a. RNA oligonucleotides used for DCL3 dicing assays**

| **Name** | **Sequence** | **Relevant Figure(s)** |
| --- | --- | --- |
| **oligos for testing DCL3 dicing of blunt or overhanging ends** | | |
| 37 nt top | 5'- ACAAGCGAAUGAGUCAUUCAUCCUAAGUCCAACAUAG -3' | 1C,5 |
| 37 nt bottom | 5'- CUAUGUUGGACUUAGGAUGAAUGACUCAUUCGCUUGU -3' | 1C, 5 |
| 38 nt bottom | 5'- CUAUGUUGGACUUAGGAUGAAUGACUCAUUCGCUUGUA -3' | 1C,5 |
| 39 nt bottom | 5'- CUAUGUUGGACUUAGGAUGAAUGACUCAUUCGCUUGUAG -3' | 1C |
| **oligos for testing DCL3 preferences for overhang length** | | |
| 38 nt top | 5'- ACAAGCGAAUGAGUCAUUCAUCCUAAGUCCAACAUAGU -3' | 1D, 1E, S1B |
| 38 nt bottom | 5'- ACUAUGUUGGACUUAGGAUGAAUGACUCAUUCGCUUGU -3' | 1E |
| 39 nt bottom | 5'- ACUAUGUUGGACUUAGGAUGAAUGACUCAUUCGCUUGUU -3' | 1D, S1B |
| 40 nt bottom | 5'- ACUAUGUUGGACUUAGGAUGAAUGACUCAUUCGCUUGUUU -3' | 1D, S1B |
| **oligos for testing DCL3 cleavage of 24/25 nt dsRNA** | | |
| 24 nt top | 5'- ACAAGCGAAUGAGUCAUUCAUCCU -3' | 2A |
| 25 nt bottom | 5'- AGGAUGAAUGACUCAUUCGCUUGUC -3' | 2A |
| **oligos for testing DCL3 cleavage of blunt-ended 23-24 nt dsRNAs** | | |
| 24 nt top | 5'- AGGAUGAAUGACUCAUUCGCUUGU -3' | 2B |
| 23 nt top | 5'- GGAUGAAUGACUCAUUCGCUUGU -3' | 2B |
| 24 nt bottom | 5'- ACAAGCGAAUGAGUCAUUCAUCCU -3' | 2B |
| **oligos for testing DCL3 cleavage of 23-24 nt dsRNAs with 3' overhangs** | | |
| 24 nt top | 5'- ACAAGCGAAUGAGUCAUUCAUCCU -3' | 2C |
| 24 nt bottom 3' overhangs | 5'- GAUGAAUGACUCAUUCGCUUGUAA -3' | 2C |
| 23 nt bottom 3' overhangs | 5'- GAUGAAUGACUCAUUCGCUUGUA -3' | 2C |
| **oligos for testing DCL3 cleavage of dsRNAs <24 nt** | | |
| 23 nt top | 5'- ACAAGCGAAUGAGUCAUUCAUCC -3' | 2D |
| 23 nt bottom | 5'- GGAUGAAUGACUCAUUCGCUUGU -3' | 2D |
| 22 nt bottom | 5'- GAUGAAUGACUCAUUCGCUUGU -3' | 2D |
| 22 nt top | 5'- ACAAGCGAAUGAGUCAUUCAUC -3' | 2D |
| **oligos for testing RNaseIII domain dicing** | | |
| 26 nt top | 5'- ACAAGCGAAUGAGUCAUUCAUCCUAA -3' | 3B, S2 |
| 27 nt bottom | 5'- UUAGGAUGAAUGACUCAUUCGCUUGUA -3' | 3B |
| 28 nt bottom | 5'- UUAGGAUGAAUGACUCAUUCGCUUGUAG -3' | S2 |
| **oligos for testing DCL3 5' nucleotide preferences** | | |
| 38 nt top 5' A | 5'- ACAAGCGAAUGAGUCAUUCAUCCUAAGUCCAACAUAGU -3' | 4A |
| 39 nt bottom 5' A | 5'- ACUAUGUUGGACUUAGGAUGAAUGACUCAUUCGCUUGUA -3' | 4A |
| 38 nt top 5' U | 5'- UCAAGCGAAUGAGUCAUUCAUCCUAAGUCCAACAUAGU -3' | 4A |
| 39 nt bottom 5' U | 5’- ACUAUGUUGGACUUAGGAUGAAUGACUCAUUCGCUUGAA -3' | 4A |
| 38 nt top 5' C | 5’- CCAAGCGAAUGAGUCAUUCAUCCUAAGUCCAACAUAGU -3’ | 4A |
| 39 nt bottom 5' C | 5’- ACUAUGUUGGACUUAGGAUGAAUGACUCAUUCGCUUGGA -3’ | 4A |
| 38 nt top 5' G | 5’- GCAAGCGAAUGAGUCAUUCAUCCUAAGUCCAACAUAGU -3’ | 4A |
| 39 nt bottom 5' G | 5’- ACUAUGUUGGACUUAGGAUGAAUGACUCAUUCGCUUGCA -3’ | 4A |
| **oligos for testing DCL3 3' nucleotide preference** | |  |
| 38 nt top 5' A | 5'- ACAAGCGAAUGAGUCAUUCAUCCUAAGUCCAACAUAGU -3' | 4B |
| 39 nt bottom 3' A | 5'- ACUAUGUUGGACUUAGGAUGAAUGACUCAUUCGCUUGUA -3' | 4B |
| 39 nt bottom 3' U | 5'- ACUAUGUUGGACUUAGGAUGAAUGACUCAUUCGCUUGUU -3' | 4B |
| 39 nt bottom 3' C | 5'- ACUAUGUUGGACUUAGGAUGAAUGACUCAUUCGCUUGUC -3' | 4B |
| 39 nt bottom 3' G | 5'- ACUAUGUUGGACUUAGGAUGAAUGACUCAUUCGCUUGUG -3' | 4B |
| **oligos for testing DCL3 5' phosphate group preferences** | | |
| 39 nt top | 5'- AACAUGUUGGACUUAGGAUGAAUGACUCAUUCGCUUGUU -3' | 4C |
| 39 nt bottom 5' triphosphate | 5'^PPP^- ACAAGCGAAUGAGUCAUUCAUCCUAAGUCCAACAUGUUU -3' | 4C |
| 39 nt bottom | 5'- ACAAGCGAAUGAGUCAUUCAUCCUAAGUCCAACAUGUUU -3 | 4C |

**Supplementary File 1b. Oligonucleotides used for Pol IV and RDR2 transcription assays**

| **Name** | **Sequence** | **Related to Figure(s)** |
| --- | --- | --- |
| **RNA oligo used for Pol IV-RDR2 transcription reaction** | | |
| 16 nt RNA primer | 5'- UGCUUUUUGUCCUGGC -3' | 6B, 6D |
| **DNA oligos used for Pol IV-RDR2 transcription reaction** | | |
| 51 nt T-less template DNA | 5’CAAAAACGAGACAGACAACGAAAGCAGACAGAGAACGCCAGGACCGACACG -3’ | 6B, 6D |
| 28 nt non-template DNA | 5’- GCTGCTTTCGTTGTCTGTCTCGTTTTTG -3’ | 6B, 6D |
| **RNA oligo used for recombinant RDR2 transcription reaction** | | |
| 37 nt template RNA | 5'- UGCGUGCGUCUGCGUCGUUCGUCCUUUGUCCUUCUUC3'^dideoxy^ | 6C |
